# Terminating a Child’s Life? Religious, Moral, Cognitive, and Emotional Factors Underlying Non-Acceptance of Child Euthanasia

**DOI:** 10.5334/pb.341

**Published:** 2017-04-26

**Authors:** Csilla Deak, Vassilis Saroglou

**Affiliations:** 1 Université catholique de Louvain, BE; 2 Belgian National Fund for Scientific Research, BE

**Keywords:** conservatism, euthanasia, moral foundations, prosociality, religion

## Abstract

Is opposition to child euthanasia motivated only by ideology, or also by other personality characteristics and individual differences? In Belgium, the first country to legalize child euthanasia (in 2014), we investigated religious, moral, emotional, and cognitive factors underlying the (dis)approval of this legalization (*N* = 213). Disapproval was associated with religiousness, collectivistic morality (loyalty and purity), and prosocial dispositions, in terms of emotional empathy and behavioral generosity, but not values (care and fairness). It was also associated with low flexibility in existential issues and a high endorsement of slippery slope arguments, but not necessarily low openness to experience. A regression analysis showed that in addition to religiousness, low flexibility in existential issues and high empathy and generosity distinctly predicted opposition to child euthanasia. Whereas most of the findings parallel those previously reported for adult euthanasia, the role of prosocial inclinations in predicting moral opposition seems to be specific to child euthanasia.

Though socially and morally controversial, euthanasia- of humans or animals- has been practiced for centuries across cultures (McDougall & Norman, 2007). Recently, in a few countries in the world, euthanasia of human adults has been decriminalized with the law in these countries specifying conditions under which this practice is permitted. Belgium is one of these few countries: In 2002, euthanasia, defined as a deliberate life-ending act by another person at the patient’s request, became legal. Twelve years later, in 2014, Belgium became the first country in the world to legalize child euthanasia, that is, to extend the possibility of legal euthanasia to minors, without any age limit—whereas, technically, in the Netherlands, euthanasia of minors is legally not extended under the age of 12. Both adult and child euthanasia are generally accepted in Belgian society, with the latter being supported by about 74% of the population (before the law was passed; Laporte, 2013).

Even if largely accepted within Belgium, non-negligible variability still exists in the attitudes of acceptance versus disapproval of the legalization of child euthanasia (e.g., Roelands, Van den Block, Geurts, Deliens, & Cohen, 2015). There are also individuals, groups, or scholars who publicly criticize this law or some of its aspects (e.g., Siegel, Sisti, & Caplan, 2014). The present work focuses on the psychological individual differences that may explain this variability. More precisely, it aims to investigate the religious, moral, emotional, and cognitive characteristics that distinguish law opponents from the societal majority; or, in correlational terms, characteristics that are related to, and may predict (in a regression), high versus low disapproval of child euthanasia. There are three broad questions of particular interest here. First, does the disapproval of child euthanasia mostly/exclusively reflect ideological preferences such as religious beliefs and moral preferences, or does it (also) reflect subtle psychological, cognitive and emotional, factors? Moreover, are psychological characteristics of moral opponents the same for adult and child euthanasia, or does the minor status of the child play a role? Finally, does prosocial orientation underline the approval of child euthanasia for alleviating suffering or, on the contrary, the disapproval of it for preserving the innocents’ right to life? The present work is the first, to our knowledge, to examine the above questions, and it does so in Belgium, a society where child euthanasia is accepted by a majority of the population. Below we will present these moral and religious, as well as cognitive and emotional, dispositions and develop the corresponding hypotheses.

## Religious Ideology

We first expected disapproval of the legalization of child euthanasia to be typical of people high in religiosity. Religious worldviews usually emphasize the idea that the world and the life of each living being are created by God, and God is solely responsible for the termination of each life. Endorsing (literally) this idea is thus likely to imply opposition to euthanasia in general, including child euthanasia (Gill, 1998). In the past (Grell & Cunningham, 2007), and sometimes today in fundamentalist communities, religions have occasionally been, or may still be, reluctant to accept any medical procedure on the human body (Vess, Arndt, Cox, Routledge, & Goldenberg, 2009).

Previous research has indeed shown that religiosity typically relates to low acceptance of adult euthanasia, both within and across countries, be they religious or secular countries (for national studies: Aghababaei, 2014; Deak & Saroglou, 2015; Stolz et al., 2015; for international studies in Europe and beyond: Cohen et al., 2006; Verbakel & Jaspers, 2010), as well as across time within countries (Danyliv & O’Neill, 2015; Jaspers, Lubbers, & De Graaf, 2007). Interestingly, Catholic individuals and countries are less accepting of euthanasia than Protestant individuals and countries, very likely because the former are generally more traditional (Verbakel & Jaspers, 2010). Note also that, across time, there is some polarization in Europe, with most of Western Europe becoming more permissive and most of Eastern Europe becoming less permissive of euthanasia (Cohen, Van Landeghem, Carpentier, & Deliens, 2014). These trends can be explained as resulting from, respectively, a growing secularization process in the former countries, and the intensification of traditional and identity affirmation-oriented forms of religion in the latter countries following their exit from atheist communist regimes. Overall, religions have become more liberal with regard to euthanasia (Moulton, Hill, & Burdette, 2006), and one study conducted in the UK even reported religious acceptance of euthanasia (Hains & Hulbert-Williams, 2013). Nevertheless, attenuation does not mean cessation; and the basic reason of religious opposition to euthanasia mentioned above (the literal belief in God as the supreme master of each being’s life) remains central in religious faith.

Our expectation of religiousness being associated with a discomfort with child euthanasia is not simply an extension of the theoretical and empirical evidence on adult euthanasia, but is based on additional and subtler psychological reasons. In fact, child euthanasia may represent a stronger moral dilemma for the religious person than adult euthanasia, which typically implicates elderly persons. Given previous theory and evidence attesting consistent links between religiosity and just-world beliefs (Jost et al., 2014), it may be that religious people find the termination of an “innocent” minor’s life particularly unjust. At the same time, the prosocial orientation – in attitudes, values, and dispositions – of religious persons, at least toward ingroup members and non-immoral persons (Saroglou, 2006, 2013, for reviews), may, on the contrary, cause discomfort for religious persons in front of an “innocent” child’s extreme suffering, and could potentially allow them to be more open to the idea of euthanasia as the only solution to alleviate such suffering. Thus, child euthanasia may put the religious person in a strong moral dilemma where compassion toward a terribly and incurably suffering “innocent” child conflicts with the moral duty to preserve human life. Indeed, research in recent years has suggested that, generally, when moral deontology is in conflict with prosocial concerns, religious people may not be at ease with deciding what is best due to their endorsement of both interpersonal and non-interpersonal morality (Deak & Saroglou, 2016; Johnson et al., 2016; Malka, Soto, Cohen, & Miller, 2011). Nevertheless, the existing evidence suggests that religiosity is typically more strongly related to deontological morality than interpersonal morality (Piazza & Sousa, 2014; Schmitt & Fuller, 2015). Consequently, we favored the hypothesis that religiosity would relate to opposition to child euthanasia. This would hold in Belgium, the country where this study was conducted. Although Belgium is a high scoring secular Western country, it has a strong Catholic tradition, which, as mentioned above, would favor the religious opposition hypothesis.^1^

## Collectivistic Moral Orientation

Behind, but also beyond, the role of religious ideology, we also expected the disapproval of child euthanasia to be related to a strong endorsement of conservative-traditional morality, that is, in terms of the Moral Foundations Theory (Haidt & Graham, 2007), collectivistic moral foundations. These foundations include *loyalty* to the group, respect for *authority*, and *purity*, that is, valuing the preservation of the natural and sacred order of things and the world. These three values constitute what Haidt and colleagues call “binding” morality, a moral orientation favoring society over individuals. These values are highly endorsed in collectivistic cultural contexts, and by conservative people in individualistic cultural contexts (Graham, Haidt, & Nosek, 2009).

Thus, disapproval of child euthanasia may stem from loyalty, that is, the strong endorsement by all members of society of the obligation to participate and contribute, through respective and reciprocal duties, to the society’s existence, cohesion, and development: No member is allowed to simply be discharged. They may also disapprove of this modern, liberal practice simply out of respect for traditional norms as expressed by various sources of societal authority. Finally, and importantly, purity is also likely to be involved, because terminating a person’s life, in particular a child’s life, through medical technology created by humans, can be perceived as violating the natural order of things and the world.

Previous research has shown the role played by the above-mentioned “binding” collectivistic morality in the opposition to the moral liberalization of various issues related to sex, family, and individual life, such as divorce, abortion, homosexuality, and suicide, as they interfere with the preservation of social groups (Deak & Saroglou, 2015; Koleva, Graham, Iyer, Ditto, & Haidt, 2012; Rottman, Kelemen, & Young, 2014). Two recent studies have shown that this also includes adult euthanasia (Deak & Saroglou, 2015, purity and loyalty; Koleva et al., 2012, purity).

Note that religiosity can be perceived to inherently include the endorsement of the three binding moral foundations (Graham & Haidt, 2010), and has indeed been found to predict their strong endorsement, in particular purity (Deak & Saroglou, 2016; Jonhson et al., 2016). Thus, it may be that religiosity and collectivistic morality share a significant common variance in predicting the disapproval of child euthanasia.

## Other-Oriented Emotions and Motives

Beyond the role of religious ideology and conservative/collectivistic moral orientation, one can argue that other-orientated motives may relate to the acceptance or disapproval of child euthanasia. Facing the possibility of alleviating extreme and hopeless suffering of a minor, by allowing or accelerating his/her death to occur, raises the following question: do people with strong other-oriented motives tend to favor euthanasia or disapprove of it? These prosocial inclinations may involve values (endorsing the importance of caring for and not harming others), emotions (feeling empathy for another person’s unfortunate situation), and behavioral inclinations (actively showing selfless generosity). We investigated this issue while being open to two competing hypotheses.

On the one hand, it can be argued that the disapproval of the legal possibility of child euthanasia due to strong deontological, collectivistic moral reasons (“we should not allow or help, under any circumstances, individuals to end their life”) also indicates weak other-oriented dispositions in values, emotions, and behavior, because the other person’s extreme suffering is neglected. Inversely, tolerance of child euthanasia should denote a capacity to empathize with others’ pain and a strong endorsement of care-based morality. Indeed, though it has been argued that conservatives value both “binding”, collectivistic morality and interpersonal morality, including the moral foundations of care and fairness (Graham, Haidt, & Nosek, 2009), there is evidence showing that the former morality may in some cases even come *at the detriment* of interpersonal morality (e.g., Piazza & Sousa, 2014; Van Pachterbeke, Freyer, & Saroglou, 2011).

In favor of this hypothetical direction (disapproval of child euthanasia is stronger among people low in prosociality), some previous research indicates that opposition to at least adult euthanasia is fueled solely by a strong emphasis on collectivist morality (in particular, loyalty and purity), and does not reflect an emphasis on interpersonal morality (care and fairness) or empathy (Deak & Saroglou, 2015; Koleva et al., 2012). This evidence could thus be extended to child euthanasia.

On the other hand, it can be argued that the decision to end a child’s life, even if for good reasons, is difficult for someone who is highly empathetic and ascribes high importance to the values care and fairness. Compared to an elderly person, a seriously ill child has not yet experienced the richness of life. Not surprisingly, people are less favorable of child than adult euthanasia (Stolz et al., 2015), and grief after the loss of a child is more complicated than other kinds of bereavement (e.g., Neidig & Dalgas-Pelish, 1991). Terminating a child’s life eliminates even the smallest chance for him/her to have positive future experiences. Thus, the disapproval of child euthanasia, in contrast to adult euthanasia, may be stronger among people with high other-oriented motives as reflected in their prosocial values, emotions, and behavior.

In favor of this second hypothesized direction (disapproval of child euthanasia is stronger among highly prosocial people), some research indicates that, at least among Iranian Muslims, agreeableness and self-reported altruism are positively associated with the rejection of adult euthanasia (Aghababei, 2014). Note that, however, in this study, this was also the case for conscientiousness, which suggests that the associations of both big five factors with opposition to euthanasia may reflect social conformity- see Bègue et al., 2015- rather than necessarily empathy, compassion, and valuing no harm. Another argument in favor of a positive link between prosocial dispositions and the disapproval of child euthanasia may be that strong concerns for children’s capacity for discernment, typical among the skeptics (Bovens, 2015; Siegel et al., 2014), and subsequent fears of possible abuses of legalization (Karlsson, Strang, & Milberg, 2007), reflect high perspective taking, an important component of empathy.

## Socio-Cognitive Characteristics: Low Flexibility

Beyond religious ideological, moral, and emotional factors, the opposition to child euthanasia may be based on socio-cognitive factors indicating low cognitive flexibility. It can be expected that such opposition reflects (1) a low openness to novelty and complexity, and thus low autonomy in thought, (2) a low relativism and readiness to question one’s own beliefs and values, independently of their content, and (3) a strong tendency to use excessively generalized risk-based rhetoric. We investigated the above expectations through the use of three constructs, respectively, openness to experience, existential quest, and slippery slope thinking style. Below we detail the rationale for our hypothesis for each of the three constructs.

Accepting the legalization of child euthanasia is something both new and complex: It requires acceptance of exceptions to the “do not kill” rule and a more nuanced understanding of this moral imperative as not being exclusively conceivable in “black-and-white” terms. Initial evidence shows that, at least among Iranian Muslims, opposition to euthanasia is related, among other things, to low openness to experience (Aghababei, 2014). Similarly, greater acceptance of euthanasia in European countries is associated with the belief in the right to self-determination (Cohen et al., 2006) and the value placed on autonomy, at both the individual and the national levels (Verbakel & Jaspers, 2010). We thus expected the disapproval of child euthanasia to be negatively related to *openness to experience*.

However, it can be argued that the disapproval of child euthanasia does not result from a generalized low flexibility across all life domains, but from low flexibility in a specific domain, that is, existential questions and worldviews. For instance, some research has shown that religious fundamentalists are not necessarily low in integrative complexity of thought in general, but only with respect to the religious/existential and the moral domains (Pancer, Jackson, Hunsberger, Pratt, & Lea, 1995). Similarly, Conway et al. (2016) recently found that conservatives are not necessarily simple-minded across all life domains: while liberals show higher complexity in certain domains (e.g., premarital sexuality, alcohol consumption, abortion), conservatives demonstrate higher complexity in other domains (e.g., death penalty, open-door immigration, loud music). We investigated this question by focusing on the construct of *existential quest*. In line with the concept of religious quest, as elaborated by Batson et al. (1993), researchers have recently defined existential quest as the readiness to question one’s own beliefs and worldviews, and to do so independently of their content, be it religious or not (Van Pachterbeke et al., 2012). We thus hypothesized that opponents of child euthanasia would show low levels of existential quest, which was indeed found to be the case for adult euthanasia (Deak & Saroglou, 2015).

Finally, we expected the disapproval of child euthanasia to be strongly related to the endorsement of a *slippery slope thinking style*. In general, slippery slope arguments are those where a premise, such as an action or a reform, must be rejected as bad (or accepted as good) on the basis of similar cases with further consequences that are much more negative (or positive) (Jefferson, 2014; Walton, 2015). In the moral and social domains, these kinds of arguments are typically used to oppose a proposed reform by linking it to a chain of disastrous consequences alleged to inevitably follow the passage of a reform: “if we do A, at some point, the highly undesirable B will follow” (Jefferson, 2014, p. 672). In particular, with regard to euthanasia, it is often feared that the acceptance of voluntary adult euthanasia for highly serious reasons will gradually extend to an acceptance of involuntary euthanasia and euthanasia of minors, as well as euthanasia for less serious reasons, or even for eugenic motives (see Jones, 2011; Verbakel & Jaspers, 2010). Psychological research has started to identify the mechanisms that contribute to the perceived strength of slippery slope arguments, such as a high similarity perceived between the beginning and the end of a slippery slope argument (Corner, Hahn, & Oaksford, 2011) or the use of inflammatory language inducing anger (Quraishi et al., 2014).

Slippery slope arguments are typically questionable at both the logical and empirical levels for being, for instance, excessive, illogical or unrealistic. Note though that they are not necessarily, always, or totally fallacious. In fact, they must be evaluated on a case-by-case basis, both logically and empirically, especially within the context of social developments (Jefferson, 2014; Walton, 2015). For instance, conservative fears that adult euthanasia would extend to minors turned out to be true; and similar fears that the acceptance of gay and lesbian civil unions would progressively lead to an acceptance of gay and lesbian adoption also turned to be true in several countries.

## Summary of the Hypotheses

In summary, we expected the disapproval of child euthanasia in Belgium – a Western secularized country of Catholic tradition – where this practice has been legalized and is rather socially accepted but still debated, to be related to, and predicted (in a regression) by, ideological factors (religious beliefs and collectivistic moral orientation), socio-cognitive tendencies (low openness to experience and existential quest, and a high use of slippery slope thinking style), and other-oriented emotions and motives (high or low prosocial dispositions: empathy, interpersonal moral principles, and behavioral intentional generosity).

## Method

### Participants

The study was advertised on online social networks as an investigation of opinions and attitudes concerning child euthanasia. Data were collected in the spring of 2015, months after the Belgian euthanasia law was extended to minors. In total, 218 participants completed the questionnaire. Five people were removed from the analyses based on their answers to “catch-items” of the Moral Foundation Questionnaire (Graham et al., 2011) that detect those who are not really paying attention to the survey. The final sample contained 213 young adults and older adults (83% women) aged 17 to 75 years (*M* = 25.5; *SD* = 10.3). The majority (79%) was Belgian, 14.2% were French, and the remaining participants were from other Western countries. (The non-Belgian participants did not differ from the Belgian participants on any of the measures.) Participants were mostly university students (76.4%). Concerning religious affiliation, the sample consisted of 40% atheists or agnostics, 36% Christians, 7% Jews, 5% Muslims, and 1% Protestants; the remaining participants had “other” affiliations.

### Measures

#### Disapproval of child euthanasia

At the beginning of the questionnaire, information was provided on the Belgian legislation of child euthanasia and the specific conditions in which euthanasia is legally permitted. Participants read the following text: “On the 3rd of March 2014, Belgium became the first country to legalize euthanasia without age limit for children and adolescents. The latter, for euthanasia to be allowed, should have an incurable disease and face unbearable suffering. Before the decision, a psychologist must ensure the child’s capacity for discernment, that is to say, his/her ability to fully comprehend all the consequences of the decision. According to the parliamentarians, the law on euthanasia does not substitute palliative care. In all cases, a discussion among healthcare professionals, as well as parental consent, is mandatory”.

The degree of endorsement of, versus opposition to, the legalization of child euthanasia was assessed with the following item: “How much do you agree with the legalization of child euthanasia?” Additionally, the degree of endorsement of, versus opposition to, the specific legal conditions for child euthanasia to be permitted was assessed by the following item: “How much do you agree with the legal conditions under which child euthanasia is practiced?” Respondents rated each item on a 6-point Likert scale ranging from 1 = *not at all agree* to 6 = *totally agree*. For both items, scores were reversed so that higher scores denote opposition. Afterwards, participants were asked to give an argument to justify their response to the first question. These spontaneous responses (some participants provided one argument, some provided more than one) were coded by two independent coders to form broad categories of the type of arguments used.

#### Hypothesized correlates

***Slippery slope thinking.*** We created four items to measure the belief that the misuse of euthanasia, legalized for exceptional cases, would lead to a generalized practice and the trivialization of the act of terminating a life: “Do you think that the legalization of child euthanasia crossed a line, and that it will be misused?”; “Do you have the impression that this decision was made because of economic reasons rather than to prevent suffering?”; “Do you think it is possible that, following this child euthanasia law, there will be other laws concerning euthanasia of the handicapped, people with dementia, the elderly, prisoners, …?”; “Do you think that this law on child euthanasia is just a step further in the growing disrespect for human life?” Participants had to rate their answers on 7-point Likert scales (α = .83).

***Spontaneous generosity.*** Participants were asked to indicate what they would do if they won 100,000 Euros, specifying each expenditure and the percentage of money they would allocate to each (see Clobert & Saroglou, 2013). The total percentage of money participants spontaneously allocated to others (e.g., family, friends, and charities) instead of themselves was coded as a score of spontaneous prosocial behavioral intention.

***Existential quest.*** The Existential Quest Scale (Van Pachterbeke, Keller, & Saroglou, 2012) assesses flexibility in existential beliefs and worldviews, that is, valuing doubt and being open to questioning and changing one’s own existential beliefs and worldviews. Sample items are: “Being able to doubt one’s convictions and reappraise them is a good quality” and “I know perfectly well what the goal of my life is (reverse)”. This is a 9-item measure with 7-point Likert scales, but we did not include two items referring specifically to religion to avoid any overlap with attitudes regarding religion (α = .66).

***Openness to experience.*** We used the 10 items of the openness to experience subscale of the Big Five Inventory (John, Naumann, & Soto, 2008; e.g., “you are creative and have a lot of original ideas”) with response scale ranging from 1 (*not at all*) to 5 (*very much*) (α = 74).

***Moral foundations.*** The 20-item Moral Foundations Questionnaire-short version (Graham et al., 2011; our French translation) was administered. This questionnaire measures the endorsement of the five moral foundations, that is, care, fairness, loyalty, authority, and purity (6-point Likert scales). Following previous work (e.g., Napier & Luguri, 2013), we also combined the care and the fairness items into a single variable that we called “interpersonal morality”, and the items of authority, loyalty, and purity into a single score representing “collectivistic morality” (respective αs = .53 and .79).

***Empathy.*** Eight items, four for empathetic concern and four for perspective taking, were selected from the Interpersonal Reactivity Index (Davis, 1983) to keep the questionnaire within a reasonable length (5-point Likert scales were used). A global score of empathy was computed by averaging the scores of the eight items (α = .67).

***Religiosity and spirituality.*** We administered three items measuring the importance of God in life, the importance of religion in life, and the frequency of prayer (a typical index of religiosity), and one item measuring the importance of spirituality in life (7-point Likert scales, except for the frequency of prayer rated on a 5-point Likert scale). Given the high intercorrelation between religiosity and spirituality, and that separately the two indexes did not provide additional information, we combined the four items into one construct that we labeled “religiousness” (α = .94).^2^

## Results

Means and standard deviations of the disapproval of child euthanasia and the disapproval of the related legal conditions, as well as of the hypothesized correlates are detailed in Table 1. On the basis of scores ≤ 3 on the 6-point Likert scale, it turned out that 71.8% of participants accepted, in various degrees, child euthanasia and 79.3% found the legal conditions acceptable. These two opinions were highly interrelated, *r* = .75, *p* < .001.

**Table 1:** Means and Standard Deviations of All Measures and Coefficients of Correlations Between the Target Variables and the Hypothesized Correlates.

			Disapproval of	

Variables	*M*	*SD*	Child Euthanasia	Legal Conditions

Disapproval of				
Child euthanasia	2.74	1.51	–	–
Legal conditions	2.55	1.62	–	–
Interpersonal morality	4.63	0.55	–.11	–.07
Care	4.38	0.71	–.08	–.09
Fairness	4.88	0.65	–.10	–.03
Collectivistic morality	3.40	0.72	.19**	.16*
Loyalty	3.29	0.77	.19**	.17*
Authority	3.43	0.87	–.01	–.06
Purity	3.47	1.03	.27***	.26***
Slippery slope thinking	3.02	1.45	.71***	.76***
Existential quest	5.04	0.90	–.25***	–.22**
Openness to experience	3.56	0.59	.03	.03
Religiousness	2.74	1.94	.52***	.56***
Empathy	4.14	0.55	.10	.03
Spontaneous generosity (%)	23.78	26.83	.29**	.26**

*p < .05. **p < .01. ***p < .001.

In the open-ended question asking for a justification of their approval or disapproval of child euthanasia, participants spontaneously used various types of arguments. Half of the 213 participants approved of child euthanasia for a prosocial motive, that is, to end the severe suffering of seriously ill children. More than one fourth of participants (28%) used a principlistic justification for their endorsement of child euthanasia by defending the right to choose one’s own death. One fifth of participants accepted child euthanasia only if the legal conditions are respected (20%). Finally, the justification most commonly used against child euthanasia was doubt regarding a child’s capacity to understand what death means and to make such an important decision (16% of the total number of participants). This justification was followed by the opposition to legal “killing” (5%) and slippery slope-like arguments (1.9%).

We carried out correlational analyses on the relationships between the disapproval of child euthanasia and its legal conditions and the individual differences variables (see Table 1). Disapproval of both child euthanasia in general, and its legal conditions in particular, were positively related to collectivistic morality, particularly to the endorsement of the moral foundations of loyalty and purity. No relationship was found between either care or fairness (interpersonal morality) and the disapproval of child euthanasia or its legal conditions. Moreover, both indicators of disapproval were strongly and positively related to slippery slope thinking as well as to religiousness. Finally, both the disapproval of child euthanasia and the disapproval of its legal conditions were negatively related to existential quest and positively related to spontaneous generosity.

Several of the hypothesized explanatory individual differences were inter-correlated (see Table 2). Empathy was meaningfully related (positively) to interpersonal morality and existential quest, but not to collectivistic morality and religiosity. On the contrary, spontaneous generosity was unrelated to empathy, existential quest, and interpersonal morality, but was positively related to collectivistic morality and religiosity. Slippery slope thinking was found to be in contrast with existential quest: the two were negatively interrelated and were, respectively, positively versus negatively associated with religiosity and its moral correlate, that is, collectivistic morality. Finally, openness to experience was characteristic of those who did not value authority as a moral foundation.

**Table 2:** Correlations between Hypothesized Predictors of Disapproval of Child euthanasia and Legal Conditions.

	Collect. morality	Slippery slope th.	Exist. quest	Open-ness	Religi-ousness	Empathy	Spont. generos.

Interper. morality	.10	–.11	.09	.02	–.12^†^	.30***	.10
Care		–.11	.04	.03	–.09	.22**	.08
Fairness		–.07	.11	.00	–.12^†^	.27***	.08
Collect. morality		.19**	–.13^†^	–.07	.31***	–.07	.25***
Loyalty		.15*	–.12^†^	.08	.31***	–.01	.20**
Authority		–.01	–.08	–.21**	.11	–.12^†^	.08
Purity		.30***	–.12^†^	–.03	.32***	–.04	.32**
Slippery slope think.			–.18**	.05	.53***	.01	.25***
Existential quest				.09	–.14*	.26***	–.08
Openness					.07	.10	.06
Religiousness						.02	.22**
Empathy							.08

^†^*p* < .10. *p < .05. **p < .01. ***p < .001.

Given the above-mentioned inter-correlations, it could be that some of these variables share common variance in predicting, in a regression, the disapproval of child euthanasia. For instance, religiousness and collectivistic morality have much in common, both theoretically and empirically, and thus it is unclear whether opposition to child euthanasia is predicted distinctly and additively by each of the two, or whether one of the two may be a confound. Moreover, it is of interest to examine whether individual differences in the (dis)approval of child euthanasia are exclusively due to the religious and moral ideologies people endorse, or also originate from subtler psychological, emotional and cognitive factors.

To clarify these issues, we conducted a hierarchical regression analysis of the disapproval of child euthanasia on the hypothesized correlates and predictors. In Step 1, religiousness was entered as a unique predictor, given past research indicating its predominant role. In Step 2, collectivistic morality (loyalty combined with purity) was added, to check for the religiosity-conservative morality overlap. In Step 3, all other individual differences variables were additionally entered: interpersonal morality (care and fairness), empathy, and spontaneous generosity, as well as openness to experience and existential quest. Slippery slope thinking was not included because it was conceptually and empirically (*r* = .71) too proximal to the disapproval of child euthanasia. At the final Step 4, age and gender were entered to control for their possible role as moderators or confounds. All VIFs were ≤ 1.3, indicating no risk of multicollinearity.

The results (see Table 3) showed that religiousness was the strongest predictor of the disapproval of child euthanasia. Although slightly decreased after the inclusion of collectivistic morality and other variables, the effect of religiousness, remained the most important. Beyond this effect, the disapproval of child euthanasia was additionally and distinctly predicted by high spontaneous generosity, high empathy, and low existential quest. Collectivistic morality was no longer a significant predictor, very likely due to its overlap with religiosity. Finally, gender and age did not impact the results. Overall, 35% of the variance was explained.

**Table 3:** Hierarchical Regression of Disapproval of Child Euthanasia on the Hypothesized Predictors.

	β	*SE*	b*	*t*-test	95% CI

Step 1					
Religiousness	0.40	0.05	.51	8.46***	[.30, .49]
*R*^2^ = .26					
Step 2					
Religiousness	0.36	0.05	.47	7.27***	[.27, .46]
Collectivistic morality	0.21	0.13	.11	1.67	[–.04, .45]
*R*^2^ = .27; Δ^2^ = .01					
Step 3					
Religiousness	0.32	0.05	.41	6.37***	[.22, .41]
Collectivistic morality	0.12	0.13	.06	0.96	[–.13, .37]
Openness to experience	–0.01	0.15	–.00	–0.03	[–.30, .29]
Existential quest	–0.31	0.10	–.19	–3.08**	[–.51, –.11]
Empathy	0.44	0.17	.16	2.58**	[.10, .78]
Spontaneous generosity	0.09	0.00	.17	2.83**	[.01, .02]
Interpersonal morality	–0.29	0.17	–.11	–1.72	[–.62, .04]
*R*^2^ = .35; Δ^2^ = .08					
Step 4					
Religiousness	0.32	0.05	.40	6.24***	[.22, .41]
Collectivistic morality	0.12	0.13	.06	0.95	[–.13, .37]
Openness to experience	–0.03	0.15	–.01	–0.21	[–.33, .27]
Existential quest	–0.30	0.10	–.18	–2.93**	[–.50, –.10]
Empathy	0.47	0.18	.17	2.71**	[.13, .82]
Spontaneous generosity	0.01	0.00	.17	2.81**	[.01, .02]
Interpersonal morality	–0.27	0.17	–.10	–1.59	[–.61, .07]
Gender	–0.21	0.25	–.05	–0.83	[–.69, .28]
Age	0.00	0.01	.02	0.29	[–.02, .02]
*R*^2^ = .35; Δ^2^ = .00					

**p < .01. ***p < .001. *Notes. Dfs* = 208. Collectivistic morality includes loyalty and purity.

## Discussion

Euthanasia of minors is an emerging issue of social and moral debate in Western countries, with Belgium being the first country in the world to legally allow, since 2014, voluntary euthanasia of minors without any age limit. The present study, using a convenience adult sample, confirmed that the acceptance of child euthanasia in Belgium is high (72%; mean disapproval = 2.74 on a 6-point Likert scale). This acceptance rate is similar to that found in a survey conducted prior to the law’s vote (74%, 2,714 Belgian adults; Laporte, 2013). However, it is slightly lower than that of adult euthanasia (86%) found in a previous study in the same country (mean disapproval = 2.54 on the same 6-point scale; Deak & Saroglou, 2015; gender balanced sample), a difference very likely due to the child’s age status. The two major arguments spontaneously provided by participants to defend child euthanasia, that is, avoiding suffering (50%) and the right to choose one’s own moment of death (28%), were the same leading justifications in a previous study in Belgium on adult euthanasia (Roelands et al., 2015). Nevertheless, in that study, the two justifications were present in reverse proportions, respectively 30% and 63%, likely due to the age of the target.

### The Predominant Role of Religiousness

Importantly, from an individual differences perspective, though the acceptability of child euthanasia was high, there was still a significant inter-individual variability. Religiousness was the strongest correlate of negative attitudes toward child euthanasia. The effect (*r* = –.52) was similar in size to the association between religiousness and the disapproval of adult euthanasia in a previous study in the same country (*r* = –.53; Deak & Saroglou, 2015). Importantly, religiousness remained a distinct and, at the same time the strongest, predictor of the disapproval of child euthanasia, even after taking into account, in the hierarchical multiple regression, the preferences for collectivistic morality and ingroup prosociality (both related to religiousness), as well as the tendency for low flexibility in worldviews. These results confirm the key role of religion in the moral opposition to several domains of modern moral liberalization (e.g., divorce, abortion, gay marriage and adoption). Of interest to note is that the role of religiousness in moral opposition has also been attested at the collective, country, level, with secularization importantly diminishing society’s opposition to these issues (e.g., Cohen et al., 2014, and Danilyv & O’Neill, 2015, for euthanasia; van den Akker, van der Ploeg, & Scheepers, 2013, for homosexuality-related issues).

The distinct effect of religiousness, beyond that of moral foundations, can also be understood in terms of partial independence between moral conviction and religious conviction, beyond their strong links (Skitka, Bauman, & Lytle, 2009). Other research has also shown that religious ideologies function as sources of beliefs that influence moral judgment, and this independently from, or even in opposition with, what humans consider to be universally moral (Turiel & Neff, 2000). This mainly concerns here the belief in God as the unique creator, and thus as the unique legitimate terminator of human life. The partial independence between religion and morality can also be seen from another perspective: religious people may think morally independent from their religion. Indeed, an inspection, in the present data, of the answers provided by those affiliated with a religion showed that 37% (most often Jews, but also 25% of the Catholics) *approved* the legalization of child euthanasia (see also Roelands et al., 2015, for similar trends regarding adult euthanasia).

### The Additional Role of Moral, Cognitive, and Emotional Factors

In addition to the role of religiousness, other results underlined the role of moral (in correlations, not in the regression), as well as cognitive and emotional factors. Those who disapproved, compared to those who accepted the legalization of child euthanasia, tended to be lower in socio-cognitive flexibility on existential issues (existential quest) and higher in collectivistic moral orientation (loyalty and purity). Interpersonal moral values (care and justice) were unrelated to moral opposition. The findings suggest that the lower flexibility involved here may not generalize to any domain, but is specifically located within the ideological, existential-moral domain. Indeed, openness to experience, a broad personality factor, did not relate to high or low acceptance; it was existential quest that predicted low opposition. This extends previous research showing that conservatives’ low flexibility does not generalize across all domains, but is specific to certain ones (Conway et al., 2015; Pancer et al., 1995).

These results importantly replicate and extend, from adult to child euthanasia, the results of the above-mentioned previous Belgian study on the psychological correlates and predictors of attitudes toward adult euthanasia (Deak & Saroglou, 2015). However, additional findings of the present study depart from those of the previous study which was focused on similar hypothetical correlates of adult euthanasia in Belgium (Deak & Saroglou, 2015). In that study, prosocial dispositions were found to be generally irrelevant in explaining the tolerance or disapproval of adult euthanasia. On the contrary, in the present work focused on child euthanasia, both empathy (in the regression) and spontaneous generosity (in both the correlational and regression analyses) turn out to be associated with the disapproval of child euthanasia. These findings provide initial evidence in favor of only one of the two possible directions developed in the introduction: People with prosocial dispositions – at least, in terms of emotional and behavioral inclinations – tend to disapprove the legalization of euthanasia (possibly because of the horror of prescribing death to an “innocent” minor even if for good reasons). The present results suggest that the alternative hypothesis, that they should endorse it in order to alleviate extreme suffering, may be wrong.

The fact that these findings are specific to child euthanasia and do not seem to extend to adult euthanasia precludes the interpretation that prosocial people are against euthanasia *in general*, and this for *altruistic, other-oriented*, motives. It is the minor status of the target that makes, in the case of child euthanasia, the moral conflict between “do not kill” and “do not let the other suffering” even stronger, allowing prosocial concerns to play their role. One meaningful interpretation is that the present findings may highlight the role of some past evolutionary, *ingroup, prosocial motives for the preservation of offspring*, kinship, and the human species. Interestingly, in the task we used to measure participants’ inclination to generosity, the beneficiaries spontaneously mentioned were mostly proximal people and ingroup members (family, friends) and not charity organizations and unknown people in need. In addition, similar to what has been previously reported (Deak & Saroglou, 2015), a strong willingness to share hypothetical gains with (proximal) others was, in the present study, related only to collectivistic, but not interpersonal morality, as well as to religiousness. Religiousness is known to typically relate to both high reproduction (Pew Research Center, 2015; Rowthorn, 2011) and a high level of prosociality mainly toward ingroup members (Galen, 2012; Saroglou, 2006). In sum, prosocial inclinations predicting opposition to child euthanasia can be better interpreted as indicating ingroup preservation motives rather than other-oriented concerns.

Also of interest to note is that it is unclear why, out of the three collectivistic moral foundations, loyalty and purity, but not authority, were related to the disapproval of child euthanasia. This is, however, consistent with previous research on adult euthanasia (Deak & Saroglou, 2015; Koleva et al., 2012). Loyalty represents the moral imperative of reciprocity within the group and the society, thus making euthanasia, be it in childhood or late adulthood, appear as a kind of treachery or as a non-assumption of one’s own responsibilities. Purity represents the need for (literal) respect and preservation of the physical and sacred order of things; thus, any intervention to terminate an individual’s life is prohibited. Respect for authority may be less relevant for understanding the (dis)approval of euthanasia. Alternatively, given the high acceptance rate of (child) euthanasia in Belgium, it could be that the role of respect for authority as a moral foundation is unclear here: does this value lead to respect of the current norm (permissiveness) or to attachment to the traditional norm (prohibition)?

Finally, the slippery slope argumentation style turned out to be very highly correlated with the disapproval of euthanasia; and the two shared the same correlates. This underlines the decisive role of this thinking style in shaping the non-acceptance of (child) euthanasia—or, alternatively, in a posteriori legitimizing moral opposition. To some extent, these slippery slope arguments, even if excessive, may be realistic. For instance, the legalization of adult euthanasia in Belgium, where this study was conducted, was indeed followed, some years later, by the legalization of child euthanasia. Future research should investigate whether moral opposition is based not only on slippery slope arguments that are specific to the moral issue under study (i.e., child euthanasia), but also on a more general style of slippery slope reasoning that is content-free.

### Limitations and Future Directions

The sample was predominantly female. It can thus not be guaranteed that the findings would apply equally to men, particularly with respect to the results on empathy and generosity. However, given that most of the results were very similar to those of a previous study on adult euthanasia in the same country, where the gender was well-balanced and did not impact the results (Deak & Saroglou, 2015), there are no strong reasons to doubt the overall generalizability of the present findings across genders. Also, only two items were used to measure the disapproval of the legalization of child euthanasia. Nevertheless, given the theoretical and empirical continuity of the present findings with previous research, the results can be viewed with confidence.

Finally, an idea for future research could be to examine the importance of just-world beliefs in causing a lower acceptability of child, compared to adult, euthanasia. Contrary to seriously ill persons of old-age who are naturally closer to death, children and adolescents have not yet fully experienced life. The option to terminate their life may thus seem to be more unfair and unjust, in turn making the decision more difficult to make. This is not only because someone will die (high tribute) without having lived his/her life (no benefit), but also because minors may be perceived as too innocent to experience a death they do not deserve. Given well-established empirical links between religion and just-world beliefs (Jost et al., 2014), this kind of belief, typical of the theodicy problem, may also significantly fuel religious opposition to child euthanasia.

## Footnotes

Regarding religious moral opposition, another interesting comparison is that between abortion and child euthanasia. In both cases, an important concern, from a religious perspective, is to not intervene in God’s power regarding human life, especially that of an “innocent” non-adult, the fetus also being considered a human person. However, abortion differs from child euthanasia. The religious opposition to the former may reflect, at least indirectly, some additional punitive tendency toward sexually liberal women when perceived as unwilling to assume their responsibilities with regard to the consequences of their sexual acts (see, e.g., Bryan & Freed, 1993).

We additionally included a measure of death anxiety (Templer, 1970). Since the termination of a child’s life is a particularly disturbing event implying stronger and more complex grieving processes, we investigated whether people with high death anxiety may tend to be reluctant toward child euthanasia. For instance, death reminders have been found to increase people’s attachment to the social group, family, and offspring (Echebarria Echabe & Saioa Perez, 2016; Fritsche et al., 2007). However, in the present study, death anxiety turned out to be entirely unrelated to the disapproval of child euthanasia and thus will not be further discussed.
